# Bis(1,3-dibenzyl­imidazolium) μ-oxido-bis­[trichloridoferrate(III)]

**DOI:** 10.1107/S1600536810024098

**Published:** 2010-06-26

**Authors:** E. M. Mutambi, C. J. Adams, A. G. Orpen

**Affiliations:** a University of Bristol, School of Chemistry, Cantock’s Close, Bristol BS8 1TS, England

## Abstract

In the title compound (C_17_H_17_N_2_)_2_[Fe_2_Cl_6_O], obtained from the solid-state reaction of FeCl_2_ and *N*,*N*′-dibenzyl­imidazolium chloride, the complex anion has approximate *D*_3*d*_ symmetry with crystallographically imposed inversion symmetry coincident with the bridging μ-O atom. The stereochemistry about each FeCl_3_O centre is distorted tetra­hedral [Fe—Cl = 2.2176 (5)–2.2427 (5) Å and Fe—O = 1.7545 (2) Å]. The Cl atoms are involved in weak anion–cation C—H⋯Cl inter­actions, giving a network structure.

## Related literature

For literature relating to the intended product, see: Yoshida *et al.* (2005 ▶); Zhong *et al.* (2007 ▶). For literature relating to anions see: Molins *et al.* (1998 ▶); Kohn *et al.* (1996 ▶); Vasilevsky *et al.* (1988 ▶).
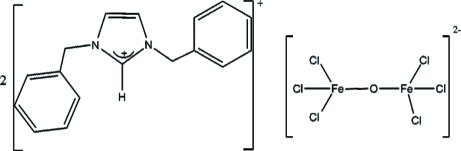


## Experimental

### 

#### Crystal data

(C_17_H_17_N_2_)_2_[Fe_2_Cl_6_O]
*M* *_r_* = 839.05
Orthorhombic, 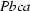

*a* = 16.2468 (5) Å
*b* = 12.8841 (4) Å
*c* = 17.6041 (5) Å
*V* = 3684.99 (19) Å^3^
*Z* = 4
Mo *K*α radiation
μ = 1.26 mm^−1^
*T* = 100 K
0.31 × 0.21 × 0.16 mm

#### Data collection

Bruker SMART CCD area-detector diffractometer
Absorption correction: multi-scan (*SADABS*; Sheldrick, 1996 ▶) *T*_min_ = 0.681, *T*_max_ = 0.815
42672 measured reflections
5399 independent reflections
4111 reflections with *I* > 2σ(*I*)
*R*_int_ = 0.052

#### Refinement

*R*[*F* ^2^ > 2σ(*F* ^2^)] = 0.033
*wR*(*F* ^2^) = 0.081
*S* = 1.04
5399 reflections
214 parameters
H-atom parameters constrained
Δρ_max_ = 0.77 e Å^−3^
Δρ_min_ = −0.44 e Å^−3^

### 

Data collection: *SMART* (Bruker, 2007 ▶); cell refinement: *SAINT* (Bruker, 2007 ▶); data reduction: *SAINT*; program(s) used to solve structure: *SHELXL97* (Sheldrick, 2008 ▶); program(s) used to refine structure: *SHELXTL* (Sheldrick, 2008 ▶); molecular graphics: *SHELXTL*; software used to prepare material for publication: *SHELXTL*.

## Supplementary Material

Crystal structure: contains datablocks I, global. DOI: 10.1107/S1600536810024098/zs2046sup1.cif

Structure factors: contains datablocks I. DOI: 10.1107/S1600536810024098/zs2046Isup2.hkl

Additional supplementary materials:  crystallographic information; 3D view; checkCIF report

## Figures and Tables

**Table 1:** Hydrogen-bond geometry (Å, °)

*D*—H⋯*A*	*D*—H	H⋯*A*	*D*⋯*A*	*D*—H⋯*A*
C1—H1⋯Cl1^i^	0.95	2.82	3.6579 (16)	147
C3—H3⋯Cl1	0.95	2.81	3.4853 (17)	129
C11—H11*A*⋯Cl1^i^	0.99	2.85	3.7440 (18)	151
C13—H13⋯Cl2^ii^	0.95	2.86	3.5619 (18)	131
C15—H15⋯Cl2^iii^	0.95	2.91	3.8403 (18)	167
C2—H2⋯Cl3	0.95	2.91	3.8223 (17)	162

Symmetry codes: (i) 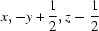; (ii) 
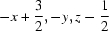
; (iii) 
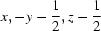
.

**Fig. 1.**
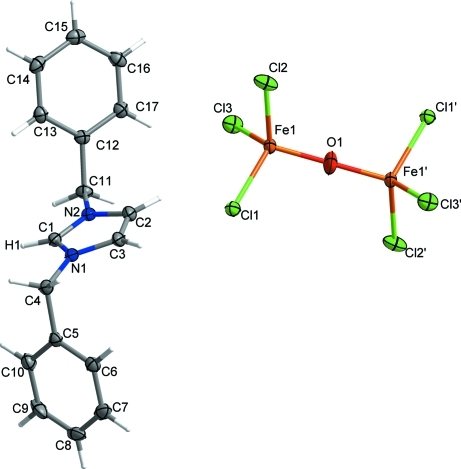
Molecular structure and atom numbering scheme for the IBz cation and the complex dianion in (I). The dianion lies across an inversion centre: for primed atoms, the symmetry code is -x + 1, -y, -z + 2. Non-H atoms are drawn at the 50% probability level.

**Fig. 2.**
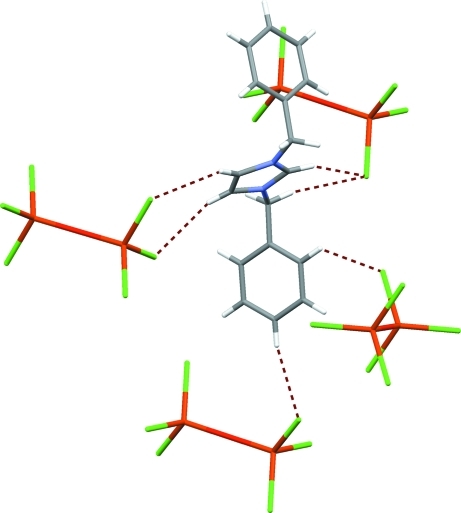
Cation–anion C—H···Cl interactions in (I).

**Fig. 3.**
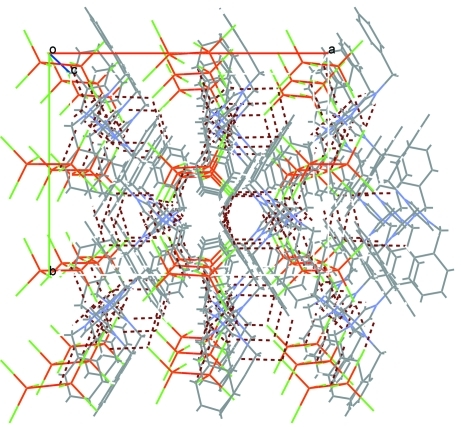
Crystal packing showing the three-dimensional networks formed in (I).

## supplementary crystallographic information

### Comment

We sought to widen the synthetic routes for the synthesis of [FeCl_4_]^-^–[HIBz]^+^ compounds (IBz = *N*,*N'*-dibenzylimidazole) by using solid state reactions without resorting to solution methods. The aim was to form the salts by grinding together the reactants and this was done with FeCl_2_ and IBz.HCl resulting in formation of the title compound (C_17_H_17_N_2_)_2_ [Cl_6_Fe_2_O] (I), which was obtained during the recrystallization process after extracting with dichloromethane and the structure is reported here. The asymmetric unit of (I) contains one HIBz^+^ cation and half of [Fe_2_OCl_6_]^2-^ dianion has *D*_3_*_d_* symmetry with crystallographically imposed inversion symmetry coincident with the bridging O atom (Fig. 1). The stereochemistry about each FeCl_3_O centre is tetrahedral [Fe–Cl, 2.2176 (5)–2.2427 (5) Å: Fe–O, 1.7545 (2) Å]. The Fe—O bond distance compares very well with the average bond length of 1.758 Å for similar anions reported in the CSD. However, the Fe—Cl bond distances are slightly longer compared to the mean Fe—Cl distance of 2.181 Å in the tetrahedral FeCl_4_, but agree well with the average distance of 2.214 Å reported in the CSD for anions similar to (I). In the cation the phenyl rings lie approximately perpendicular to the plane of the HIBz ring [torsion angles C1–N1–C4–C5, 86.90 (19)°; C1–N2–C11–C12, 109.17 (18)°].

The crystal structure shows weak cation–anion C—H···Cl hydrogen-bonding interactions (Table 1, Fig. 2), giving a network structure (Figure 3).

### Experimental

FeCl_2_ (0.0299 g: 0.1 mmol) and *N*,*N'*-dibenzylimidazolium chloride (0.0858 g: 0.2 mmol) were ground together for *ca.* 10 minutes using a mortar and pestle. A small amount of the crystalline product was dissolved in dichloromethane and allowed to slowly evaporate at room temperature, giving after 3 days, yellow plate-like crystals of (I) suitable for X-ray diffraction.

### Refinement

H atoms were positioned geometrically and refined using a riding model with C—H = 0.93 Å (aromatic) or 0.99 Å (aliphatic) and U_iso_(H) = 1.2 times U_eq_ (C, N).

### Crystal data

**Table:**

(C_17_H_17_N_2_)_2_[Fe_2_Cl_6_O]	*F*(000) = 1712
*M**_r_* = 839.05	*D*_x_ = 1.512 Mg m^−^^3^
Orthorhombic, *P**b**c**a*	Mo *K*α radiation, λ = 0.71073 Å
Hall symbol: -P 2ac 2ab	Cell parameters from 8129 reflections
*a* = 16.2468 (5) Å	θ = 2.3–29.0°
*b* = 12.8841 (4) Å	µ = 1.26 mm^−^^1^
*c* = 17.6041 (5) Å	*T* = 100 K
*V* = 3684.99 (19) Å^3^	Plate, yellow
*Z* = 4	0.31 × 0.21 × 0.16 mm

### Data collection

**Table:**

Bruker SMART CCD area-detector diffractometer	5399 independent reflections
Radiation source: sealed tube	4111 reflections with *I* > 2σ(*I*)
graphite	*R*_int_ = 0.052
Detector resolution: 9.091 pixels mm^-1^	θ_max_ = 30.1°, θ_min_ = 3.4°
φ and ω scans	*h* = −22→22
Absorption correction: multi-scan (*SADABS*; Sheldrick, 1996)	*k* = −10→18
*T*_min_ = 0.681, *T*_max_ = 0.815	*l* = −24→24
42672 measured reflections	

### Refinement

**Table:**

Refinement on *F*^2^	Primary atom site location: structure-invariant direct methods
Least-squares matrix: full	Secondary atom site location: difference Fourier map
*R*[*F*^2^ > 2σ(*F*^2^)] = 0.033	Hydrogen site location: inferred from neighbouring sites
*wR*(*F*^2^) = 0.081	H-atom parameters constrained
*S* = 1.04	*w* = 1/[σ^2^(*F*_o_^2^) + (0.0331*P*)^2^ + 1.977*P*] where *P* = (*F*_o_^2^ + 2*F*_c_^2^)/3
5399 reflections	(Δ/σ)_max_ = 0.001
214 parameters	Δρ_max_ = 0.77 e Å^−^^3^
0 restraints	Δρ_min_ = −0.44 e Å^−^^3^

### Special details

**Table:**

Geometry. All e.s.d.'s (except the e.s.d. in the dihedral angle between two l.s. planes) are estimated using the full covariance matrix. The cell e.s.d.'s are taken into account individually in the estimation of e.s.d.'s in distances, angles and torsion angles; correlations between e.s.d.'s in cell parameters are only used when they are defined by crystal symmetry. An approximate (isotropic) treatment of cell e.s.d.'s is used for estimating e.s.d.'s involving l.s. planes.
Refinement. Refinement of *F*^2^ against ALL reflections. The weighted *R*-factor *wR* and goodness of fit *S* are based on *F*^2^, conventional *R*-factors *R* are based on *F*, with *F* set to zero for negative *F*^2^. The threshold expression of *F*^2^ > σ(*F*^2^) is used only for calculating *R*-factors(gt) *etc*. and is not relevant to the choice of reflections for refinement. *R*-factors based on *F*^2^ are statistically about twice as large as those based on *F*, and *R*- factors based on ALL data will be even larger.

### Fractional atomic coordinates and isotropic or equivalent isotropic displacement parameters (Å^2^)

**Table:**

	*x*	*y*	*z*	*U*_iso_*/*U*_eq_	
Fe1	0.572901 (17)	0.002174 (18)	0.926492 (14)	0.01778 (7)	
N1	0.69781 (9)	0.34450 (10)	0.70045 (8)	0.0158 (3)	
N2	0.60555 (9)	0.24363 (10)	0.65187 (8)	0.0162 (3)	
C1	0.65919 (11)	0.31864 (12)	0.63681 (9)	0.0168 (3)	
H1	0.6684	0.3489	0.5883	0.020*	
C2	0.60972 (11)	0.22098 (13)	0.72855 (9)	0.0191 (3)	
H2	0.5782	0.1704	0.7550	0.023*	
C3	0.66719 (11)	0.28445 (13)	0.75867 (9)	0.0191 (3)	
H3	0.6835	0.2872	0.8105	0.023*	
C4	0.75812 (11)	0.42987 (12)	0.70917 (9)	0.0180 (3)	
H4A	0.7904	0.4374	0.6618	0.022*	
H4B	0.7967	0.4132	0.7509	0.022*	
C5	0.71438 (11)	0.53029 (12)	0.72638 (9)	0.0158 (3)	
C6	0.70433 (11)	0.56215 (13)	0.80122 (10)	0.0192 (3)	
H6	0.7271	0.5220	0.8413	0.023*	
C7	0.66112 (12)	0.65228 (14)	0.81769 (11)	0.0255 (4)	
H7	0.6553	0.6744	0.8689	0.031*	
C8	0.62652 (12)	0.70995 (14)	0.75939 (12)	0.0283 (4)	
H8	0.5958	0.7708	0.7706	0.034*	
C9	0.63691 (13)	0.67870 (14)	0.68495 (12)	0.0289 (4)	
H9	0.6136	0.7186	0.6450	0.035*	
C10	0.68116 (12)	0.58936 (14)	0.66809 (10)	0.0226 (4)	
H10	0.6887	0.5688	0.6167	0.027*	
C11	0.54927 (11)	0.19507 (13)	0.59652 (10)	0.0182 (3)	
H11A	0.5494	0.2364	0.5491	0.022*	
H11B	0.4926	0.1961	0.6173	0.022*	
C12	0.57281 (11)	0.08439 (12)	0.57817 (9)	0.0159 (3)	
C13	0.64112 (11)	0.06417 (13)	0.53308 (9)	0.0185 (3)	
H13	0.6745	0.1199	0.5158	0.022*	
C14	0.66090 (12)	−0.03689 (14)	0.51314 (9)	0.0209 (4)	
H14	0.7082	−0.0503	0.4829	0.025*	
C15	0.61153 (12)	−0.11859 (13)	0.53742 (10)	0.0219 (4)	
H15	0.6244	−0.1877	0.5228	0.026*	
C16	0.54355 (12)	−0.09921 (14)	0.58294 (10)	0.0213 (4)	
H16	0.5102	−0.1551	0.5999	0.026*	
C17	0.52421 (12)	0.00244 (13)	0.60382 (10)	0.0184 (3)	
H17	0.4780	0.0157	0.6354	0.022*	
Cl1	0.63474 (3)	0.15767 (3)	0.93098 (2)	0.02198 (10)	
Cl2	0.66715 (3)	−0.12117 (3)	0.94427 (3)	0.02821 (11)	
Cl3	0.51889 (3)	−0.02728 (3)	0.81283 (3)	0.02493 (10)	
O4	0.5000	0.0000	1.0000	0.0421 (6)	

### Atomic displacement parameters (Å^2^)

**Table:**

	*U*^11^	*U*^22^	*U*^33^	*U*^12^	*U*^13^	*U*^23^
Fe1	0.02114 (14)	0.01791 (12)	0.01430 (12)	−0.00244 (9)	0.00495 (10)	0.00096 (8)
N1	0.0180 (8)	0.0140 (6)	0.0153 (6)	0.0019 (5)	0.0011 (6)	−0.0013 (5)
N2	0.0190 (8)	0.0135 (6)	0.0160 (6)	0.0021 (5)	0.0017 (6)	−0.0007 (5)
C1	0.0204 (9)	0.0156 (7)	0.0144 (7)	0.0025 (6)	0.0018 (7)	0.0000 (6)
C2	0.0234 (10)	0.0164 (8)	0.0174 (8)	0.0015 (7)	0.0040 (7)	0.0035 (6)
C3	0.0252 (10)	0.0170 (8)	0.0151 (7)	0.0038 (7)	0.0017 (7)	0.0026 (6)
C4	0.0169 (9)	0.0170 (8)	0.0202 (8)	−0.0002 (6)	0.0008 (7)	−0.0017 (6)
C5	0.0147 (8)	0.0145 (7)	0.0182 (8)	−0.0022 (6)	0.0011 (6)	0.0006 (6)
C6	0.0189 (9)	0.0187 (8)	0.0200 (8)	−0.0023 (6)	0.0013 (7)	0.0001 (6)
C7	0.0246 (10)	0.0211 (9)	0.0308 (10)	−0.0048 (7)	0.0083 (8)	−0.0076 (7)
C8	0.0206 (10)	0.0137 (8)	0.0506 (12)	−0.0014 (7)	0.0110 (9)	−0.0004 (8)
C9	0.0252 (11)	0.0218 (9)	0.0397 (11)	0.0013 (7)	0.0011 (9)	0.0168 (8)
C10	0.0251 (10)	0.0222 (9)	0.0204 (8)	−0.0006 (7)	0.0022 (7)	0.0058 (6)
C11	0.0173 (9)	0.0170 (8)	0.0202 (8)	0.0001 (6)	−0.0016 (7)	−0.0014 (6)
C12	0.0179 (9)	0.0155 (7)	0.0144 (7)	0.0020 (6)	−0.0029 (7)	−0.0002 (5)
C13	0.0191 (9)	0.0206 (8)	0.0159 (7)	−0.0007 (7)	−0.0012 (7)	0.0012 (6)
C14	0.0224 (10)	0.0255 (9)	0.0148 (8)	0.0065 (7)	−0.0015 (7)	−0.0010 (6)
C15	0.0297 (11)	0.0196 (8)	0.0163 (8)	0.0058 (7)	−0.0071 (7)	−0.0024 (6)
C16	0.0250 (10)	0.0182 (8)	0.0208 (8)	−0.0020 (7)	−0.0053 (7)	0.0027 (6)
C17	0.0189 (9)	0.0203 (8)	0.0160 (7)	0.0015 (7)	−0.0016 (7)	0.0006 (6)
Cl1	0.0318 (3)	0.01609 (19)	0.01806 (19)	−0.00222 (16)	0.00283 (17)	−0.00090 (14)
Cl2	0.0343 (3)	0.0168 (2)	0.0335 (2)	−0.00020 (18)	−0.0127 (2)	0.00265 (16)
Cl3	0.0263 (2)	0.0239 (2)	0.0246 (2)	0.00011 (17)	−0.00685 (19)	−0.00070 (16)
O4	0.0488 (15)	0.0435 (13)	0.0342 (12)	−0.0089 (10)	0.0261 (11)	−0.0015 (9)

### Geometric parameters (Å, °)

**Table:**

Fe1—O4	1.7545 (2)	C7—H7	0.9500
Fe1—Cl3	2.2176 (5)	C8—C9	1.381 (3)
Fe1—Cl2	2.2290 (5)	C8—H8	0.9500
Fe1—Cl1	2.2427 (5)	C9—C10	1.389 (3)
N1—C1	1.327 (2)	C9—H9	0.9500
N1—C3	1.377 (2)	C10—H10	0.9500
N1—C4	1.481 (2)	C11—C12	1.511 (2)
N2—C1	1.328 (2)	C11—H11A	0.9900
N2—C2	1.383 (2)	C11—H11B	0.9900
N2—C11	1.476 (2)	C12—C13	1.389 (2)
C1—H1	0.9500	C12—C17	1.394 (2)
C2—C3	1.350 (2)	C13—C14	1.386 (2)
C2—H2	0.9500	C13—H13	0.9500
C3—H3	0.9500	C14—C15	1.391 (3)
C4—C5	1.507 (2)	C14—H14	0.9500
C4—H4A	0.9900	C15—C16	1.387 (3)
C4—H4B	0.9900	C15—H15	0.9500
C5—C10	1.387 (2)	C16—C17	1.396 (2)
C5—C6	1.390 (2)	C16—H16	0.9500
C6—C7	1.387 (2)	C17—H17	0.9500
C6—H6	0.9500	O4—Fe1^i^	1.7545 (2)
C7—C8	1.386 (3)		
			
O4—Fe1—Cl3	113.304 (18)	C9—C8—C7	119.78 (17)
O4—Fe1—Cl2	110.417 (18)	C9—C8—H8	120.1
O4—Fe1—Cl1	106.901 (16)	C7—C8—H8	120.1
Cl3—Fe1—Cl1	111.221 (19)	C8—C9—C10	120.49 (17)
Cl2—Fe1—Cl1	108.92 (2)	C8—C9—H9	119.8
C1—N1—C3	108.45 (14)	C10—C9—H9	119.8
C1—N1—C4	125.94 (14)	C5—C10—C9	119.86 (17)
C3—N1—C4	125.39 (14)	C5—C10—H10	120.1
C1—N2—C2	108.44 (14)	C9—C10—H10	120.1
C1—N2—C11	125.69 (14)	N2—C11—C12	112.61 (14)
C2—N2—C11	125.84 (15)	N2—C11—H11A	109.1
N1—C1—N2	108.93 (14)	C12—C11—H11A	109.1
N1—C1—H1	125.5	N2—C11—H11B	109.1
N2—C1—H1	125.5	C12—C11—H11B	109.1
C3—C2—N2	106.84 (15)	H11A—C11—H11B	107.8
C3—C2—H2	126.6	C13—C12—C17	119.72 (15)
N2—C2—H2	126.6	C13—C12—C11	120.09 (15)
C2—C3—N1	107.33 (15)	C17—C12—C11	120.14 (16)
C2—C3—H3	126.3	C14—C13—C12	120.40 (16)
N1—C3—H3	126.3	C14—C13—H13	119.8
N1—C4—C5	110.28 (14)	C12—C13—H13	119.8
N1—C4—H4A	109.6	C13—C14—C15	119.96 (17)
C5—C4—H4A	109.6	C13—C14—H14	120.0
N1—C4—H4B	109.6	C15—C14—H14	120.0
C5—C4—H4B	109.6	C16—C15—C14	120.04 (16)
H4A—C4—H4B	108.1	C16—C15—H15	120.0
C10—C5—C6	119.58 (16)	C14—C15—H15	120.0
C10—C5—C4	120.39 (15)	C15—C16—C17	120.01 (17)
C6—C5—C4	119.98 (15)	C15—C16—H16	120.0
C7—C6—C5	120.31 (17)	C17—C16—H16	120.0
C7—C6—H6	119.8	C12—C17—C16	119.86 (17)
C5—C6—H6	119.8	C12—C17—H17	120.1
C8—C7—C6	119.95 (17)	C16—C17—H17	120.1
C8—C7—H7	120.0	Fe1—O4—Fe1^i^	180.0
C6—C7—H7	120.0		
			
C3—N1—C1—N2	−0.60 (19)	C7—C8—C9—C10	0.6 (3)
C4—N1—C1—N2	−175.46 (14)	C6—C5—C10—C9	−1.2 (3)
C2—N2—C1—N1	0.37 (19)	C4—C5—C10—C9	176.32 (17)
C11—N2—C1—N1	178.63 (14)	C8—C9—C10—C5	0.8 (3)
C1—N2—C2—C3	0.01 (19)	C1—N2—C11—C12	109.77 (18)
C11—N2—C2—C3	−178.25 (15)	C2—N2—C11—C12	−72.3 (2)
N2—C2—C3—N1	−0.37 (19)	N2—C11—C12—C13	−73.19 (19)
C1—N1—C3—C2	0.60 (19)	N2—C11—C12—C17	109.41 (18)
C4—N1—C3—C2	175.50 (15)	C17—C12—C13—C14	0.4 (2)
C1—N1—C4—C5	86.90 (19)	C11—C12—C13—C14	−177.04 (15)
C3—N1—C4—C5	−87.12 (19)	C12—C13—C14—C15	0.9 (3)
N1—C4—C5—C10	−82.77 (19)	C13—C14—C15—C16	−1.4 (3)
N1—C4—C5—C6	94.71 (18)	C14—C15—C16—C17	0.6 (3)
C10—C5—C6—C7	0.2 (3)	C13—C12—C17—C16	−1.2 (3)
C4—C5—C6—C7	−177.29 (16)	C11—C12—C17—C16	176.24 (15)
C5—C6—C7—C8	1.1 (3)	C15—C16—C17—C12	0.7 (3)
C6—C7—C8—C9	−1.5 (3)		

Symmetry codes: (i) −*x*+1, −*y*, −*z*+2.

### Hydrogen-bond geometry (Å, °)

**Table:**

*D*—H···*A*	*D*—H	H···*A*	*D*···*A*	*D*—H···*A*
C1—H1···Cl1^ii^	0.95	2.82	3.6579 (16)	147
C3—H3···Cl1	0.95	2.81	3.4853 (17)	129
C11—H11A···Cl1^ii^	0.99	2.85	3.7440 (18)	151
C13—H13···Cl2^iii^	0.95	2.86	3.5619 (18)	131
C15—H15···Cl2^iv^	0.95	2.91	3.8403 (18)	167
C2—H2···Cl3	0.95	2.91	3.8223 (17)	162

Symmetry codes: (ii) *x*, −*y*+1/2, *z*−1/2; (iii) −*x*+3/2, −*y*, *z*−1/2; (iv) *x*, −*y*−1/2, *z*−1/2.
